# The Efficacy of 480 ml Oral Sodium Sulfate for Improving Insufficient Bowel Preparation of Colonoscopy with High-Concentrated Polyethylene Glycol

**DOI:** 10.1155/2023/6359165

**Published:** 2023-09-30

**Authors:** Naohisa Yoshida, Yoshikazu Inagaki, Daisuke Hasegawa, Reo Kobayashi, Yuri Tomita, Hikaru Hashimoto, Ryohei Hirose, Osamu Dohi, Ken Inoue, Yasutaka Morimoto, Yutaka Inada, Takaaki Murakami, Yoshito Itoh

**Affiliations:** ^1^Department of Molecular Gastroenterology and Hepatology, Kyoto Prefectural University of Medicine, Graduate School of Medical Science, Kyoto, Japan; ^2^Department of Gastroenterology, Nishijin Hospital, Kyoto, Japan; ^3^Department of Gastroenterology, Ayabe City Hospital, Kyoto, Japan; ^4^Department of Gastroenterology, Kyoto Saiseikai Hospital, Kyoto, Japan; ^5^Department of Gastroenterology, Kyoto First Red Cross Hospital, Kyoto, Japan; ^6^Department of Gastroenterology, Aiseikai Yamashina Hospital, Kyoto, Japan

## Abstract

**Objectives:**

Oral sulfate solution (OSS) is used for bowel preparation (BP) during colonoscopy. The way of taking this agent can be used a same-day regimen (only on the day of colonoscopy) and split regimen (the day before and on the day of colonoscopy) for receiving it. In this study, we analyzed the efficacy of a same-day regimen of 480 ml OSS for insufficient bowel preparation (BP) with high-concentrated polyethylene glycol (H-PEG).

**Materials and Methods:**

This multicenter retrospective study was conducted from December 2021 to December 2022 at three related institutions on patients aged ≥ 20 years with a fair or poor Aronchick score of BP with 1 l H-PEG in previous colonoscopy. All patients received a low-residual diet and 10 ml of 0.75% picosulfate sodium a day before the colonoscopy and 480 ml of OSS and ≥1 l of water 3 hours before the colonoscopy. We analyzed the rate of improvement with OSS compared to H-PEG and other efficacies, and adverse events (AE).

**Results:**

We evaluated 125 cases (77 males) with an average age of 72.1 ± 8.8 years. The completion rate of 480 ml of OSS was 97.6% (122/125). The improvement rate of BP showing good or excellent score with OSS was 70.4% (88/125). Compared OSS with previous H-PEG, the insertion time (min) was 7.0 ± 4.8 vs. 8.1 ± 6.0 (*p* = 0.01), and the adenoma detection rates were 67.2% vs. 63.2% (*p* = 0.05). The cleansing time (min) was 131 ± 46 vs. 165 ± 53 (*p* < 0.01). The rate of AE with OSS was 10.4% (13/125). There were no significant differences about AE in age and gender. The tolerance of OSS compared with H-PEG (good/similar/bad) was 72.0%/24.8%/3.2% (amounts), 26.4%/39.2%/34.4% (taste), and 76.8%/10.4%/12.8% (overall preference), respectively.

**Conclusions:**

The same-day regimen of 480 ml OSS effectively improved the insufficient BP of 1 l H-PEG.

## 1. Introduction

Polyp resection by colonoscopy is reported to lead to a reduction in colorectal cancer deaths [1]. However, up to 30% of colonoscopies have insufficient bowel preparation (BP), leading to decreased lesion detection due to poor visualization and an increased need for repeat colonoscopies [2, 3]. Old age, male sex, inpatient status, diabetes mellitus, constipation, and tricyclic antidepressant use are known to be associated with inadequate BP [4]. To improve insufficient BP, we regularly strengthen the BP method by increasing the amount of BP agents, changing BP agents, adding a laxative, and strict diet limitations. Regarding BP agents, polyethylene glycol (PEG) has been used widely for decades due to its good bowel-cleansing effect and safety [5, 6]. However, bowel preparation with PEG requires the administration of a large amount of the solution of up to 4 l. There is a same-day regimen and a split regimen for receiving the bowel preparation solution. The former is the way patients take it on the day of colonoscopy, and the latter is the way patients take it the day before colonoscopy and the other half on the day of colonoscopy. Several guidelines recommend split-dose bowel preparation for high-quality bowel cleansing and reduction of patient burden [5, 6]. To reduce the amount of solution and improve patient tolerability, high-concentration polyethylene glycol (H-PEG) up to 2 l was subsequently developed and has been most widely used in Japan [5, 6]. High-concentrated polyethylene glycol (H-PEG) up to the amount of 2 l was developed afterwards, and it has been most widely used in Japan [5–7]. Oral sulfate solution (OSS) was developed as a BP agent in 2010 and has been marketed in Japan since 2021. OSS contains three sulfate salts of sodium, magnesium, and potassium as active ingredients, and the amount of OSS is 980 ml (one plastic bottle: 480 ml in Japan), which is twice the amount of water required after OSS administration [8]. Several randomized control trials (RCT) have shown noninferiority about bowel preparation between OSS and H-PEG [8–10]. On the other hand, a recent meta-analysis conducting 8 RCTs showed the quality of bowel preparation in the OSS group was better than that of the PEG-based solutions group [11]. Additionally, a previous report suggested OSS could decrease the amount of BP solutions for Japanese people [12]. However, the mean total amount of OSS and water in a Japanese RCT exceeded 2 l (2384.3 ± 545.2 ml in the OSS same-day group and 2866.9 ± 86.5 ml in OSS split-dose group) [8, 12]. We previously reported the efficacy of 1 l H-PEG with a low-residual diet and 10 ml of 0.75% picosulfate sodium one day before colonoscopy to decrease the amount of H-PEG for Japanese people, and the rate of good bowel preparation exceeded 90% for 5427 Japanese people with a mean age of 64.5 ± 13.8 years old [13]. According to these papers, the bowel-cleansing effect of OSS is possibly higher than that of H-PEG, and we thought we could decrease the amount of OSS, including low-residual diet and picosulfate sodium.

In the present study, we analyzed the efficacy of a same-day 480 ml (one plastic bottle: Sulprep, Fuji Pharma Co., Ltd., Tokyo, Japan) of OSS as bowel preparation for insufficient BP with 1 l H-PEG in a previous colonoscopy.

## 2. Patients and Methods

This multicenter, single-arm, retrospective cohort study was conducted from December 2021 to December 2022 at three related institutions. The institutions were the Kyoto Prefectural University of Medicine, Nishijin Hospital, and Ayabe City Hospital. We reviewed patients aged ≥ 20 years who had a fair or poor Aronchick score of BP by 1 L of H-PEG in previous colonoscopy and received 480 ml of OSS on the day of the total colonoscopy [14]. We also reviewed patients receiving 480 ml of OSS on the day of the total colonoscopy because they had a fair or poor score by 1 l H-PEG in colonoscopy before the previous and then received 1 l H-PEG with 1-week additional 1-2 sachets of PEG (Movicol, EA Pharma, Tokyo, Japan) in previous colonoscopy according to a previous report [15]. For these patients, some received good or excellent Aronchick score due to additional sachets of PEG in the previous colonoscopy, but we used the data of poor or fair score by 1 l of H-PEG in the one before the previous colonoscopy in the current study. We analyzed the rate of improvement with OSS compared to H-PEG. The inclusion criteria were: cancer screening, positive fecal immunohistochemical examination, or surveillance after surgical or endoscopic resection of polyps and cancers. Generally, patients with fair preparation in previous colonoscopy received colonoscopy 12-24 months after previous colonoscopy and those with poor preparation received 3-6 months after it. Patients with fatal cardiopulmonary and hepatic diseases were excluded. Additionally, patients with renal dysfunction of estimated glomerular filtration rate ≤ 30 ml/min were excluded according to OSS drug information. We also excluded cases without detailed clinical data from previous colonoscopy and cases receiving partial colectomy after previous colonoscopy with 1 l H-PEG.

The evaluation items for this study were patient characteristics (age, height, body weight, and body mass index) and improvement in colonoscopic BP with OSS. We divided all cases into improved and nonimproved BP groups and analyzed the colonoscopic status and effect-related factors among patient characteristics, underlying diseases, and concomitant medications. Colonoscopic status included the rate of cecal intubation, insertion time, pain score, total procedure time, adenoma detection, and sessile serrated lesion (SSL) detection. Each operator scored the pain as 0 (no pain), 1 (mild pain), 2 (moderate pain), or 3 (severe pain). Additionally, the colonoscopic examination status was compared between 480 ml of OSS and previous H-PEG. Patients whose explanation about BP and cleansing times were calculated were examined in limited cases in both groups according to electrical medical records. Adverse events (AE) of 480 ml OSS were also examined. The tolerability of 480 ml of OSS compared to H-PEG from the patient questionnaire was evaluated in terms of amount, taste, speed, and overall preference. Patients who underwent blood chemical examinations both before and after OSS were examined.

The improvement in BP with OSS was defined as a change to good or excellent Aronchick score for all patients. With respect to the way of current and previous colonoscopic BP using OSS and H-PEG, we followed our previous report [13]. In brief, patients received a low-residual diet the day before colonoscopy and consumed 10 ml of 0.75% picosulfate sodium at 9-10 PM on the day. All patients received 480 ml of OSS and ≥1.0 l of water 3 hours before colonoscopy. For previous H-PEG, patients took 1 l of highly concentrated PEG and ≥0.5 l of water 3 hours before colonoscopy. If adequate BP was not achieved within 4 hours, 1-2 enemas were performed. All colonoscopies were performed by ten endoscopists (five experts and five nonexperts). Experts were defined as endoscopists with experience in performing more than 5,000 colonoscopies [15]. The histopathological diagnosis of lesions, including adenoma and SSL, was made according to the 2019 WHO classification [16].

Informed consent for colonoscopy was obtained from all patients prior to colonoscopy. This study was held retrospectively at all three institutions as a subgroup analysis of a multicenter prospective and retrospective study organized by our department. It was approved by the Ethics Committee of Kyoto Prefectural University of Medicine (ERB-C-1704-3, approved data: June 29, 2021) and was conducted in accordance with the World Medical Association Declaration of Helsinki. An opt-out of the study to the patients was performed in each hospital (in a website and/or on the board of an endoscopic unit).

## 3. Statistical Analyses

The Mann–Whitney *U* test, chi-square test, and Yates continuity correction were used in this study. The Mann–Whitney *U* test was used to compare the continuous variables. Categorical variables were analyzed using the chi-square test and the Yates continuity correction. All statistical analyses were performed using SPSS software (IBM Japan, Ltd., Tokyo, Japan). *p* < 0.05 was considered significant for all statistical analyses.

## 4. Results

**Figure 1:**
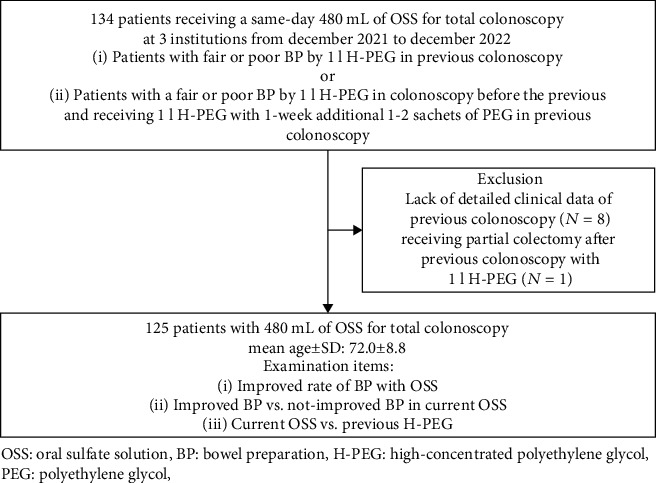
A flow diagram of the present study.

After excluding 11 patients who did not meet the inclusion criteria, we analyzed 125 patients to determine the improvement in BP (Figure 1 and Table 1). The previous BP was 1 l H-PEG for 82 patients and 1 l H-PEG+1-week sachets of PEG for 43 patients. For the 43 patients, 31 patients (72.1%) received good or excellent BP score in the last colonoscopy, but all 43 patients received fair or poor BP score only with H-PEG in the one before the last colonoscopy. The completion rate of 480 ml of OSS was 97.6% (122/125). Four cases (3.2%) received enema.

**Figure 2:**
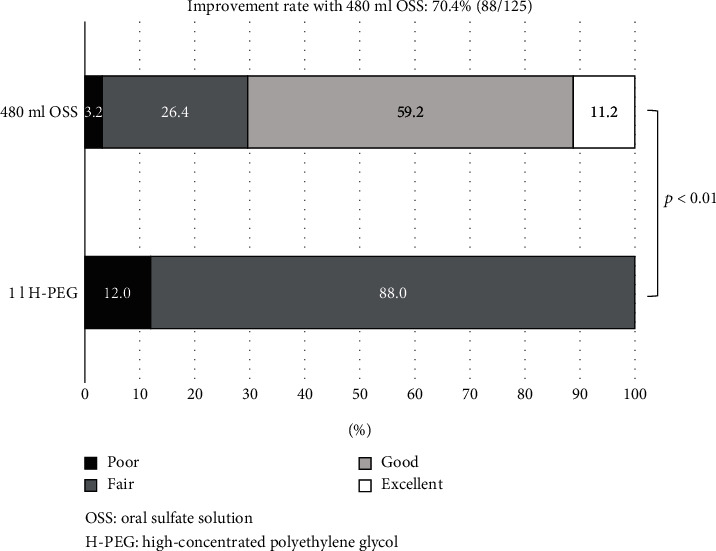
Improvement rate of bowel preparation with 480 ml OSS compared to 1 l H-PEG.

Regarding the H-PEG method, the numbers of poor/fair/good/excellent score in Aronchick score were 15/110/0/0 (12.0%/88.0%/0.0%/0.0%) (Figure 2). Those of the 480 ml OSS group were 4/33/74/14 (3.2%/26.4%/59.2%/11.2%). The status of BP (improvement/similar/worse) for all 125 patients by 480 ml OSS compared to H-PEG was 70.4%/25.6%/4.0% (88/34/5). The improvement rates of patients with H-PEG or H-PEG with additional sachets of PEG were 68.3% (56/82) and 74.4% (32/43).

A comparison between the improved and nonimproved BP groups was made (Table 2). There were no significant differences concerning age, sex, body mass index, presence of various underlying diseases, or use of concomitant drugs. Regarding colonoscopy, there was a significant difference in the insertion time (min) (6.6 ± 4.3 vs. 8.1 ± 5.5, *p* = 0.03) between the improved BP and nonimproved BP groups. Additionally, the two groups had no significant differences in adenoma and SSL detection.

A comparison between the 480 ml OSS method and H-PEG method is shown (Table 3). There were no significant differences concerning the cecal intubation rate and pain score. There were significant differences in insertion time (min) (7.0 ± 4.8 vs. 8.1 ± 6.0, *p* = 0.01) and total procedure time (min) (19.5 ± 9.8 vs. 22.6 ± 7.4, *p* < 0.01) between the two methods. There was no significant difference in SSL detection, but adenoma detection showed a marginal difference (67.2% vs. 63.2%, *p* = 0.05). There were significant differences in explanation time (sec) (392 ± 90 vs. 525 ± 150, *p* = 0.03) for 20 cases and cleansing time (min) (134 ± 46 vs. 165 ± 53, *p* < 0.01) for 30 cases between the two methods.

The AE of OSS was observed in 13 cases (10.4%): 6 cases (4.8%) of nausea, 5 cases (4.0%) of vomiting, and 2 cases (2.4%) of diarrhea (Table 4). There were no significant differences in age and gender.

The tolerances of OSS compared to H-PEG (good: similar: bad) were 72.0%: 24.8%: 3.2% (amounts), 26.4%: 39.2%: 34.4% (taste), and 76.8%: 10.4%: 12.8% (overall preference) (Table 5).

With respect to the safety of 480 ml of OSS, the details of the 44 cases before and after OSS were examined (Table 6). There were significant differences about hematocrit (HCT): 42.3 ± 3.8 vs. 43.2 ± 4.0, *p* = 0.02; creatine (mg/dL): 0.83 ± 0.21 vs. 0.88 ± 0.20, *p* < 0.01). There were no significant differences in the levels of sodium, potassium, chloride, and magnesium in the blood.

## 5. Discussion

In the current study, we demonstrated the effectiveness of 480 ml OSS for insufficient BP with 1 l H-PEG, and the improvement rate was 70.4%. The tolerability of this method was acceptable according to the patient questionnaire, and 76.8% of patients preferred 480 ml OSS to 1 l H-PEG. The insertion and procedure times decreased significantly compared to the previous 1 l H-PEG. However, adenoma and SSL detection rates did not increase significantly, although adenoma detection showed a marginal difference (*p* = 0.05).

An RCT from Korea showed the noninferior efficacy of OSS cleansing in the elderly aged 65-80 years compared to 2 l H-PEG [17]. A systematic review showed that Boston Bowel Preparation Score in the OSS group was significantly high (MD 0.32, 95% confidence interval (CI):0.03–0.62; *p* = 0.03) [18]. Another RCT compared 480 ml OSS to 1 l H-PEG, similar to our study, which showed no differences in the adequacy of BP, with 98.8% and 96.6%, respectively [19]. Compared with these studies, only patients with insufficient BP who underwent H-PEG were enrolled in our study. However, we could show another way of OSS as an alternative to H-PEG.

A systematic review of eight RCTs comparing OSS to H-PEG showed that the adenoma detection rate was significantly higher in OSS than in H-PEG (44.60% vs. 38.14%, risk ratio (RR):1.17, 95% CI:1.03-1.33, *p* = 0.01) [20]. This may be due to the high efficacy of bowel-cleansing of OSS. However, our study only showed a marginal difference in adenoma detection rates between OSS and H-PEG due to small number.

Regarding AE due to OSS, the rates were 4.0% (8/200) in the same-day dose group and 9.4% (19/202) in the split-dose group in a previous Japanese RCT, all of which were mild gastrointestinal disorders, including nausea, vomiting, nasopharyngitis, and protein urine [8]. Another study comparing OSS to H-PEG in the elderly aged 65-80 years showed that vomiting (11.6% vs. 2.1%) and thirst (24.2% vs. 11.7%) were more common in the OSS group than in the 2 l H-PEG group [17]. A systematic review of seven RCT comparing OSS to H-PEG showed that OSS was associated with a 30% increased risk of nausea (RR 1.35, 95% CI:1.03-1.77, *p* = 0.03) and more than double the risk of vomiting (RR 2.30, 95% CI:1.63-2.23, *p* < 0.05) [19]. The rate in the current study was 10.4%, and nausea and vomiting were frequent, which is similar to previous reports. We showed no significant differences in AE according to age or sex. Additionally, we could show minor dehydration after OSS so that the values of HCT significantly increased to 43.2 ± 4.0 (after OSS) from 42.3 ± 3.8 (before OSS). Electrocytes, including magnesium, did not change significantly after OSS. Another study comparing 1 l H-PEG to OSS also showed significant elevation of serum creatinine in both groups (1 l H-PEG:0.77 vs. 0.91, *p* < 0.001, OSS: 0.78 vs. 0.78, *p* = 0.04) [18]. According to these results, we suggested 480 ml OSS was a safe method for insufficient BP with H-PEG.

The cleansing time of the same-day dose of OSS in a Japanese RCT was 170.2 ± 57.4 min and which was longer than that in our study (134 ± 46 min). This was due to the difference in the amount of OSS. According to this short cleansing time in the current study, even for morning colonoscopy, patients do not have to get up early and do not disturb sleep like other BP agents and the split method. This method is also useful for urgent cases because it requires quicker BP measurement than a regularly scheduled colonoscopy. However, 480 ml of OSS was only examined in cases with insufficient BP in a previous colonoscopy. Thus, a large-scale study is required to confirm this. Additionally, the explanation time for OSS with the medical staff was shorter than that for H-PEG. Because OSS in Japan is marketed in plastic bottles, patients do not have to make solutions such as H-PEG. The sequence of OSS and water is also simple, particularly for the same-day method. We suggest that this simple method is useful, especially for the elderly, and our study included 61 patients aged ≥ 75 years.

This study was limited by its retrospective nature and a small number of cases. Thus, there was a selection bias in the enrolled patients because it was not consecutive and was decided by each doctor. Additionally, we evaluated the method of 480 ml OSS only for rescue of H-PEG. All patients examined had poor bowel preparation with H-PEG. Thus, patients and endoscopists knew that patients had poor bowel preparation with previous colonoscopy. This may have influenced the results of this study. The way of 480 ml OSS was evaluated only for Japanese people whose mean height and body weight were 162.5 ± 8.1 cm and 60.8 ± 12 kg, being smaller than Western people. Blood chemical examination data before and after OSS and cleansing/explanation time of OSS/HPEG were obtained in a limited number of cases.

## 6. Conclusion

The 480 ml OSS method as the BP of colonoscopy was effective for improving the insufficient BP of H-PEG, and this rescue method was safe regardless of age and sex.

## Figures and Tables

**Table 1:** Patients' characteristics.

	125
Age, mean ± SD	72.1 ± 8.8
Age distribution, *n* (%) ≤74: ≥75	64: 61 (51.2 : 48.8)
Gender, *n* (%) male: female	77 : 48 (61.6 : 38.4)
Height, mean ± SD (range)	162.5 ± 8.1 (143-178)
Body weight, mean ± SD (range)	60.8 ± 12.3 (36-96)
Body mass index, mean ± SD (range)	22.8 ± 3.5 (15.8-31.5)

Underlying disease	
Diverticulum, *n* (%)	46 (36.8)
Colorectal surgery, *n* (%)	34 (27.2)
Diabetes, *n* (%)	24 (19.2)
Parkinson's disease, *n* (%)	3 (2.4)

Concomitant medication	
Laxatives, *n* (%)	34 (27.2)
Antidepressants, *n* (%)	10 (8.0)

Previous BP	
1 l H-PEG: 1 l H-PEG+1-week sachets of PEG, *n* (%)	82: 43 (65.6 : 34.4)

OSS	
Reasons for selecting OSS, *n* (%) Poor or fair BP: poor or fair BP with AE of H-PEG	113 : 12 (91.4 : 9.6)
Completion of 480 ml OSS, *n* (%)	122 (97.6)

SD: standard deviation; BP: bowel preparation; H-PEG: high-concentrated polyethylene glycol; PEG: polyethylene glycol; OSS: oral sulfate solution; AE: adverse events.

**Table 2:** The comparison between cases with and without improvement of bowel preparation using 480 ml OSS.

	Improved BP	Nonimproved BP	*p* value
Case number	88 (70.4)	37 (29.6)	
Age, mean ± SD	72.1 ± 8.5	72.4 ± 9.5	0.72
Age, *n* (%), ≤74: ≥75	44 (50.0): 44 (50.0)	20 (54.1): 17 (45.9)	0.67
Gender, *n* (%), male: female	55 (62.5): 33 (37.5)	22 (59.5): 15 (40.5)	0.74
Body mass index, mean ± SD	22.9 ± 3.5	22.4 ± 3.7	0.32
Underlying disease			
Diverticulum, *n* (%)	33 (37.5)	13 (35.1)	0.80
Colorectal surgery, *n* (%)	23 (26.1)	11 (29.7)	0.68
Diabetes, *n* (%)	17 (19.3)	7 (18.9)	0.95
Parkinson's disease	1 (1.1)	2 (5.4)	0.43
Concomitant medication			
Laxatives, *n* (%)	22 (25.0)	12 (32.4)	0.39
Antidepressants, *n* (%)	9 (10.2)	1 (2.7)	0.29
Colonoscopy			
Cecal intubation rate, *n* (%)	88 (100.0)	37 (100.0)	1.0
Insertion time (min), mean ± SD	6.6 ± 4.3	8.1 ± 5.5	0.03
Pain score, mean ± SD	1.4 ± 0.6	1.5 ± 0.5	0.69
Total procedure time (min), mean ± SD	19.5 ± 6.9	19.9 ± 8.7	0.51
Adenoma detection, *n* (%)	56 (63.6)	23 (62.2)	0.93
SSL detection, *n* (%)	17 (19.3)	9 (24.3)	0.52

OSS: oral sulfate solution; SD: standard deviation; BP: bowel preparation; SSL: sessile serrated lesions.

**Table 3:** The comparison of colonoscopic status between 480 ml OSS and 1 l H-PEG.

	480 ml OSS *N* = 125	1 l H-PEG *N* = 125	*p* value
Cecal intubation rate, *n* (%)	125 (100.0)	125 (100.0)	1.0
Insertion time (min), mean ± SD	7.0 ± 4.8	8.1 ± 6.0	0.01
Pain score, mean ± SD	1.5 ± 0.7	1.4 ± 0.6	0.15
Total procedure time (min), mean ± SD	19.5 ± 9.8	22.6 ± 7.4	<0.01
Adenoma detection, *n* (%)	84 (67.2)	69 (63.2)	0.05
SSL detection, *n* (%)	26 (20.8)	17 (13.6)	0.13
Explanation time of BP with medical staff (sec), mean ± SD	392 ± 90 (*N* = 20)	525 ± 150 (*N* = 20)	0.03
Cleansing time (min), mean ± SD	134 ± 46 (*N* = 30)	165 ± 53 (*N* = 30)	<0.01

OSS: oral sulfate solution; H-PEG: highly-concentrated polyethylene glycol; SD: standard deviation; SSL: sessile serrated lesion; BP: bowel preparation.

**Table 4:** Adverse events of 480 ml OSS.

	Case number	Adverse events, *n* (%)	*p* value	Nausea, *n* (%)	Vomiting, *n* (%)	Thirsty, *n* (%)
Overall	125	13 (10.4)		6 (46.2)	5 (38.5)	2 (15.4)
≤74years old	61	7 (11.5)	0.92	3 (42.9)	3 (42.9)	1 (14.3)
≥75 years old	64	6 (9.4)		3 (50.0)	2 (33.3)	1 (16.7)
Male	77	5 (6.5)	0.13	2 (40.0)	2 (40.0)	1 (20.0)
Female	48	8 (16.7)		4 (50.0)	3 (37.5)	1 (12.5)

OSS: oral sulfate solution.

**Table 5:** Tolerability of 480 ml OSS compared to 1 l H-PEG from patients questionnaire.

	480 ml OSS *N* = 125
Amount, *n* (%) good: similar: bad	90 : 31 : 4 (72.0: 24.8: 3.2)
Taste, *n* (%) good: similar: bad	33 : 49 : 43 (26.4: 39.2: 34.4)
Cleansing speed, *n* (%) *N* = 113 fast: similar: slow	69 : 38 : 6 (61.1: 33.6: 5.3)
Overall preference, *n* (%) OSS: similar: H-PEG	96 : 13 : 16 (76.8: 10.4: 12.8)

OSS: oral sulfate solution; H-PEG: high-concentrated polyethylene glycol.

**Table 6:** The safeness of 480 ml OSS with blood chemical examination before OSS vs. after OSS.

	Before OSS *N* = 44	After OSS *N* = 44	*p* value
HCT, mean ± SD	42.3 ± 3.8	43.2 ± 4.0	0.02
CRE (mg/dL), mean ± SD	0.83 ± 0.2	0.88 ± 0.2	<0.01
Na (mEq/L), mean ± SD	140 ± 2.6	139 ± 2.6	0.07
K (mEq/L), mean ± SD	4.2 ± 0.3	4.1 ± 0.3	0.25
Cl (mEq/L), mean ± SD	103 ± 2.8	101 ± 2.6	0.07
Mg (mEq/L), mean ± SD	2.0 ± 0.1	2.0 ± 0.1	0.38

HCT: hematocrit; SD: standard deviation; CRE: creatinine; Na: sodium; K: potassium; Cl: Chloride; Mg: magnesium; OSS: oral sulfate solution.

## Data Availability

Patient data used to support the findings of this study are available from the corresponding author upon request. However, some of them are restricted by the institutional review board of the Kyoto Prefectural University of Medicine.
